# Duloxetine in OsteoArthritis (DOA) study: effects of duloxetine on pain and function in end-stage hip and knee OA – a pragmatic enriched randomized controlled trial

**DOI:** 10.1186/s12891-022-05034-0

**Published:** 2022-02-05

**Authors:** T. Blikman, W. Rienstra, T. M. van Raaij, A. J. ten Hagen, B. Dijkstra, W. P. Zijlstra, S. K. Bulstra, M. Stevens, I. van den Akker-Scheek

**Affiliations:** 1 Department of Orthopedics, University of Groningen, University Medical Center Groningen, P.O. Box 30001, Groningen, RB 9700 The Netherlands; 2 Department of Rehabilitation Medicine, University of Groningen, University Medical Center Groningen, P.O. Box 30001, Groningen, RB 9700 The Netherlands; 3 Department of Orthopedics, Martini Hospital Groningen, Groningen, The Netherlands; 4 Department of Anaesthesiology, Martini Hospital Groningen, Groningen, The Netherlands; 5 Department of Orthopedics, Medical Center Leeuwarden, Leeuwarden, The Netherlands

**Keywords:** Duloxetine, Osteoarthritis, Hip, Knee, Pain

## Abstract

**Background:**

Some osteoarthritis (OA) patients experience inadequate pain relief from analgesics like acetaminophen and nonsteroidal anti-inflammatory drugs. This could be the result of experienced non-nociceptive centralized pain. Placebo-controlled randomized trials (RCT) have proven the effectiveness of duloxetine for OA and several chronic pain conditions where central sensitization (CS) is one of the key underlying pain mechanisms.

**Objectives:**

Assess the efficacy of an 8-week duloxetine treatment compared to usual care in end-stage knee and hip OA patients with a level of centralized pain.

**Design:**

Pragmatic, enriched, open-label RCT.

**Methods:**

Patients were randomized to duloxetine or to care-as-usual. Primary outcome was pain in the index joint, measured with the pain domain of the Knee injury and Osteoarthritis Outcome Score (KOOS) or the Hip disability and Osteoarthritis Outcome Score (HOOS). The intention-to-treat principle was used, with mixed-model repeated measures to analyze the effect.

**Results:**

One hundred eleven patients were randomized. Nearly 44% felt much to very much better after duloxetine usage compared to 0% in the care-as-usual group (*p* < 0.001). The duloxetine group scored 11.3 points (95%CI: 5.8, 16.8) better on the pain domain of the KOOS/HOOS (*p* < 0.001). Knee patients improved significantly more than hip patients (18.7 [95%CI: 11.3, 26.1] versus 6.0 [95%CI: − 2.6, 14.5] points better).

**Conclusions:**

Adding duloxetine treatment seems to be beneficial for end-stage knee OA patients with neuropathic-like symptoms (at risk of CS). End stage Hip OA patients seem to be nonresponsive to duloxetine.

**Trial registration:**

Dutch Trial Registry with number NTR 4744 (15/08/2014) and in the EudraCT database with number 2013–004313-41.

**Supplementary Information:**

The online version contains supplementary material available at 10.1186/s12891-022-05034-0.

## Background

Osteoarthritis (OA) is characterized by disability and eventually invalidating pain that leads to seeking medical aid [1]. The pain experience in OA typically transitions from intermittent weight-bearing to a more persistent ongoing chronic pain [1]. Treatment is aimed at pain alleviation to regain physical function and quality of life [1]. It is known that some patients do not experience adequate pain relief from first-line treatment modalities like acetaminophen and nonsteroidal anti-inflammatory drugs (NSAIDs) [2]. This ineffectiveness probably arises from OA-related mechanopathology and the biological response to mechanically induced injury, which likely differs per individual [3]. One potential biological response is a change in the biochemical environment around peripheral joint nociceptors and joint structures [4]. This could lead to hyperexcitability of the peripheral and ultimately the central nervous system (central sensitization [CS]) [4–6].

CS is defined as an “increased responsiveness of nociceptive neurons in the central nervous system”; “this may include increased responsiveness due to dysfunction of endogenous pain control systems” [7]. It is thought that preoperative CS combined with peripheral articular nerve changes are accountable for joint-related neuropathic-like symptoms such as hyperalgesia and allodynia [8]. About 20–67% of knee OA patients and 20% of hip OA patients experience those symptoms [8–15]. Numerous studies report that these symptoms correlate strongly with basic pain intensity [8–15].

Duloxetine, a selective serotonin and norepinephrine reuptake inhibitor, seems effective in treating neuropathic pain conditions as well as chronic pain conditions where CS is one of the key underlying pain mechanisms [16–18]. In contrast to conventional OA analgesics, the mechanism of action is thought to be related to amelioration of the central pain control system by influencing serotonin and norepinephrine transporters (activation of the descending pain inhibitory system) [19].

In knee OA patients, placebo-controlled randomized trials have proven the effectiveness of duloxetine as a potent analgesic in the conservative treatment phase of OA [17, 19–22]. However, thus far studies among hip OA patients are lacking. Moreover, all previous knee OA studies were placebo-controlled so did not involve a care-as-usual control situation. Pragmatic randomized trials are needed to enhance external validity [23]. As subgroups of OA patients could react differently to analgesics, selection of a predefined group of potential responders to the treatment – in this case patients experiencing OA pain with neuropathic features – could even enhance results and reduce the number needed to treat [6]. The objective of this study is therefore to assess the efficacy of duloxetine for end-stage knee and hip OA compared to usual care in the reduction of knee- or hip-related pain by means of a pragmatic enriched randomized controlled trial. The effects on neuropathic-like symptoms, pain sensitization, physical functioning and the patient’s global impression of improvement are also assessed.

## Methods

The Duloxetine in OsteoArthritis (DOA) study was a multicenter, pragmatic, enriched, open-label randomized controlled trial aiming to assess the effects of duloxetine treatment.

### Population

The study was conducted at University Medical Center Groningen (UMCG), Martini Hospital Groningen and Medical Center Leeuwarden, the Netherlands. Patients were included between December 2014 and June 2018. Adult primary hip and knee OA patients (age > 18 years) who experienced OA pain with neuropathic features (as a sign of a centralized pain component [CS]) when placed on the waiting list for total joint arthroplasty by their orthopedic surgeon were considered eligible. Selection of a predefined group of potential responders (enriched design) was based on previous research showing that knee patients with OA pain and neuropathic features had six times higher odds of experiencing signs of CS than those with only nociceptive pain [8]. The radiological and clinical criteria for diagnosis of OA American College of Rheumatology were also used [24, 25]. Radiological criteria were checked by plain radiographs of the index joint within the previous year. Clinical criteria were checked at baseline by a researcher (T.B. or W.R.).

Patients were excluded if they underwent hip or knee joint procedures in the past year, received intra-articular injections in the past 3 months, had cognitive and/or neurological disorders that could interfere strongly with questionnaires, were likely to be hospitalized during the course of the study, were planned for total hip/knee arthroplasty (THA/TKA) within the study duration (current planned arthroplasty not included), or had significant peripheral nerve injury (e.g. polyneuropathy). Patients with previous exposure to duloxetine or duloxetine-specific contraindications were excluded. For a detailed list of duloxetine-related exclusion criteria used, see the published study protocol [26].

### Patient enrollment

**Fig. 1:**
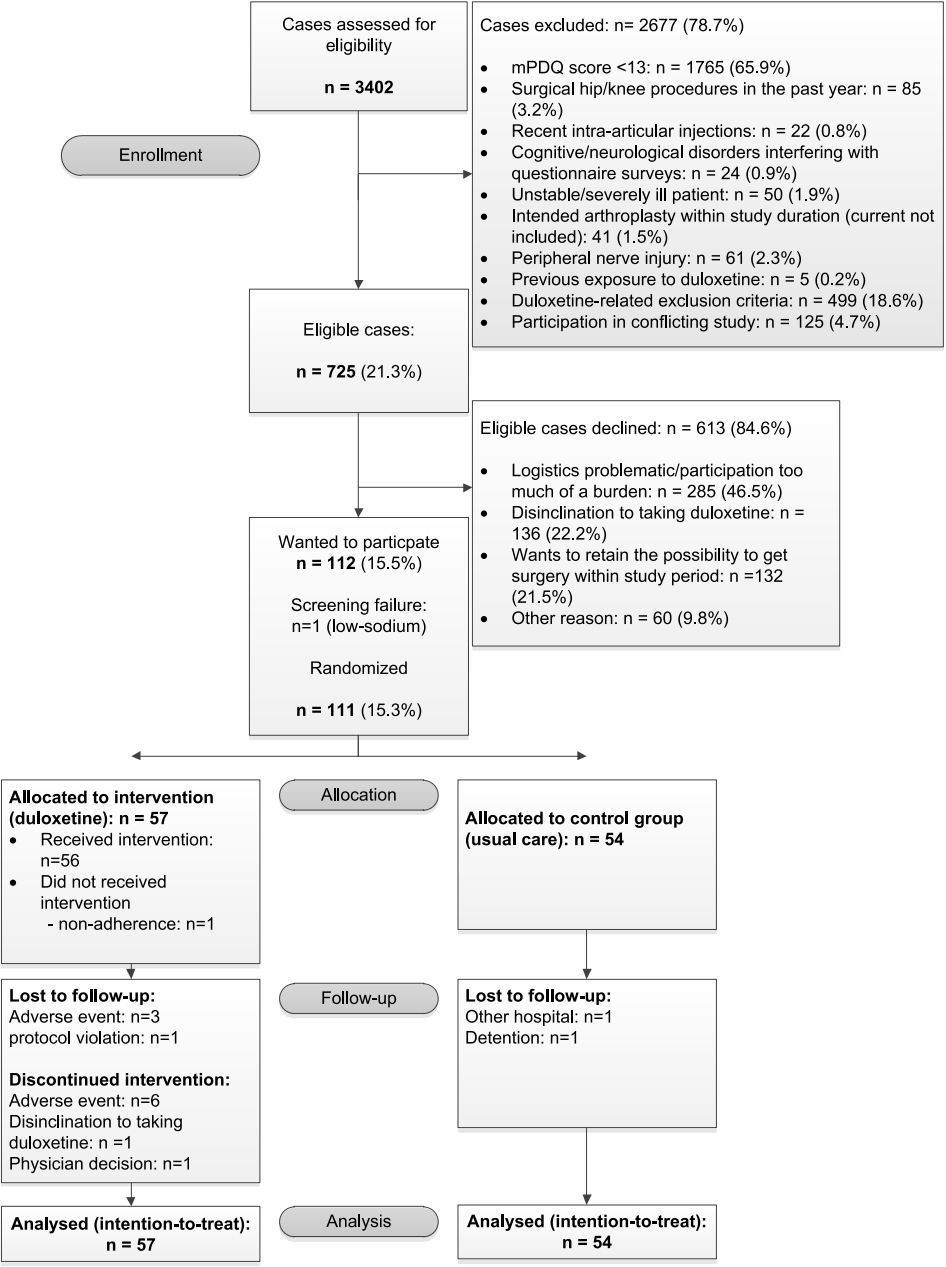
CONSORT Flow Diagram

When patients were placed on the waiting list for primary THA/TKA they were subsequently asked to fill in the modified painDETECT (mPDQ) questionnaire [11, 27]. This questionnaire asks about neuropathic-like symptoms. When a patient screened positive for OA pain with neuropathic features (mPDQ score ≥ 12) and agreed to future contact with the researcher, he/she received extensive written information and was invited to participate in the trial. See the CONSORT Flow Diagram for an overview of this process (Fig. 1). During the baseline visit (T0) all inclusion and exclusion criteria were checked and written informed consent was obtained. Baseline assessment was subsequently performed, including patient characteristics and baseline values for outcome measures (see the design paper for detailed inclusion and exclusion criteria [26]). Next, patients were randomly allocated (1:1 ratio) by means of a web-based system (ALEA, FormsVision, Abcoude, The Netherlands) to the duloxetine intervention or usual care. A stratification factor was the type of arthroplasty (hip/knee), with block sizes of 4 and 6.

### Intervention and measurement protocol

The intervention consisted of 10 weeks’ preoperative duloxetine treatment (7 weeks on target dosage). For purposes of safety and adherence, medication release took place at three different time points. Prior to medication release, the participant was again informed and warned about possible side effects. A chart was used to record usage and side effects. This chart was collected and discussed at every subsequent visit (T1, T2, T3). Time point T1 followed after medication period 1: the initiation period (weeks 1 and 2). The first week started at the day of randomization (T0), with half of the target dose (30 mg/day) to reduce the risk of side effects [28]. In the second week duloxetine was up-titrated to the target dosage of 60 mg/day. Period 2 started hereafter: the treatment phase (weeks 3 to 8), during which the target dosage of 60 mg/day was maintained. After this period, time point T2 followed. The last 2 weeks entailed period 3: the tapering phase (weeks 9 and 10). Duloxetine dosage was lowered to 30 mg/day for 2 weeks to reduce the risk of developing discontinuation symptoms [29]. The actual T3 measurement followed 4 days after the last duloxetine usage (time frame days 5–8). For the care-as-usual group T3 followed 10 weeks after baseline.

Patients who did not tolerate duloxetine discontinued the intervention and were advised to enter the tapering phase. However, tailored discontinuation advice was given to patients who discontinued at or before the treatment phase (before T1). The control group did not receive a specific intervention and solely received standard care-as-usual (any medication that was already prescribed by their physician). As it was a pragmatic RCT, no restrictions were imposed on usage of escape (pain) medication or other medication. An elaborate report of what was performed at each time point, including a scheme, is described in the published study protocol of the DOA study [26].

### Measures

#### Patient characteristics

Collected at baseline were age (at time of inclusion), gender, body mass index (BMI), cohabitation (yes/no), educational level (no or lower, secondary, higher), smoking (yes/no), ASA (American Society of Anesthesiologists) classification [30] (assessed by anesthesiologist: I, II, III or IV), comorbidities (yes/no for nine groups of diseases associated with diminished quality of life and mortality [31]: migraine, hypertension, pulmonary disease, chronic bowel disorder, severe or persistent back disorder, diabetes, myocardial infarction, severe cardiac condition, cancer), pain catastrophizing level (range 0–52 points, higher scores on the Pain Catastrophizing Scale [PCS] reflect a higher amount of experienced catastrophizing thoughts or feelings [32–34]), level of anxiety and level of depression (Hospital Anxiety and Depression Scale [HADS-Anxiety; HADS-Depression], two 7-item scales ranging 0–21 points [35]), number of painful regions/joints on most days of the previous month (body diagram, 20 regions: head, neck, shoulders, elbows, wrists, hands, upper spine, lower spine, hips, knees, ankles, feet), duration of OA pain, Kellgren and Lawrence (KL) OA-grade classification (I-IV) [36], history of surgery in index joint (yes/no), number of analgesic injections in index joint in the past year and analgesic usage in the past week.

#### Outcome measures

Primary outcome was pain in the index knee or hip, measured with the pain domain of the Knee injury and Osteoarthritis Outcome Score (KOOS) [37] or the Hip disability and osteoarthritis outcome score (HOOS) [38]. All primary and secondary endpoints were tested at time point T2, which is at the end of the treatment phase. In addition, all endpoints were tested at T3 to analyze the effect of the tapering phase. Secondary outcome measures were perceived improvements in functional status and quality of life, neuropathic-like symptoms, pressure pain sensitization, pain at rest and during movement, and the patient’s global impression of improvement. Safety measures assessed included adverse events (AE) experienced by the duloxetine intervention group.

#### Functional status and quality of life

Functional status and quality of life were measured using the KOOS [37] or HOOS [38]. The KOOS and HOOS both consist of five subscales: Pain (KOOS: 9 items; HOOS: 10 items), Other Symptoms (KOOS: 7 items; HOOS: 5 items), Activities of Daily Living (KOOS/HOOS: 17 items), Sport and Recreation (KOOS: 5 items; HOOS: 4 items), Function (KOOS: 5 items; HOOS: 4 items) and knee-related Quality of Life (KOOS/HOOS: 4 items). Standardized response options are given and each question is scored from 0 to 4 (on a 5-point Likert scale). A normalized score is subsequently calculated for each subscale ranging 0–100, with 0 indicating extreme symptoms and 100 indicating no symptoms. The Dutch version has been proven to be valid and reliable [39, 40]. Missing items were replaced where possible, by using the KOOS/HOOS manual [41, 42].

#### Neuropathic-like symptoms

Neuropathic-like symptoms were determined using the self-reported mPDQ [27] which is composed of seven items evaluating pain quality, one item evaluating pain pattern, and one item evaluating pain radiation. The total score is an aggregated score ranging from − 1 to 38. The 12-point cutoff point was used to discriminate unlikely NP phenotype patients (mPDQ≤12) from possible NP phenotype patients (mPDQ> 12). The PDQ has been validated in a heterogeneous group of low back pain patients, with 80% sensitivity and specificity (cutoff point PDQ ≥ 18, reference: two pain physicians’ diagnoses) [43]. Only one small validation study among knee OA patients was done, finding a sensitivity of 50% and a specificity of 74% for the cutoff point of > 12 (reference: quantitative sensory testing exam) [8]. The Dutch mPDQ hip/knee proved to be reliable [27] and has adequate structural and construct validity [44].

#### Pressure pain sensitization

Blunt pressure pain thresholds (PPT) were measured by an algometer (Force Ten FDX 25 Digital force gage, Wagner, instruments, Greenwich, CT, USA; 1 cm2 flat rubber tip). PPTs are proven to be highly reliable at painful, nonpainful and remote body sites [45–47]. PPTs were executed following the German Research Network on Neuropathic Pain (DFNS) protocol [48]. The test sides for the knee were the center of the patella and for the hip the greater trochanter region (5 cm distally from the greater trochanter and subsequently 2 cm anteriorly). The remote side tested was the same for all subjects: 5 cm proximally from the distal radioulnar joint (wrist area of the contralateral side). The algometer will exert force at a slowly increasing ramp of 0.5 kg/s (~ 50 kPa/s). Pressure was applied until the patient defined the pressure as slightly unpleasant (no significant painful feeling); at that point the algometer was instantly removed and the maximum force was noted. PPTs are considered to be a reflection of peripheral sensitization at the site of the joint [49]. At a remote site it is considered to reflect systemic altered pain processing/CS [49]. The PPTs at each site were assessed three times and the average of those measurements was calculated for each time point.

#### Pain during the past week

The Visual Analogue Scale (VAS) is widely used to measure pain. Pain ratings were recorded on a 100 mm horizontal line, where 100 mm represents the worst pain imaginable and zero no pain. Patients were asked to note their mean pain status at rest over the last week (VAS-R: pain at rest while sitting, standing or lying down) and during movement (VAS-M: pain during regular walking). The VAS is demonstrated as valid for measuring and comparing between chronic pain conditions [50].

#### Patient global impression of improvement

The Patient Global Impression of Improvement (PGI-I) scale measured patients’ perceived change in overall well-being (in response to therapy/treatment). It is a 7-point Likert scale that ranges from “very much worse” to “very much improved”, and is derived from the clinical global impression scale [51]. The PGI-I questionnaires were previously included as endpoints in several other duloxetine, musculoskeletal pain-related, clinical studies conducted worldwide [52–55]. Answers “much better” and “very much better” were an indicator of relevant improvement. Answers “much worse” or “very much worse” were and indicator of relevant deterioration.

### Statistical analyses

Statistical analyses were conducted by using IBM SPSS (V.23). Descriptive statistics were used to describe the study sample. Sample size was determined based on 80% power (two-sided significance level of 0.05) to detect a difference of 10 points on the KOOS/HOOS pain domain in the total study population with a standard deviation of 17.2 points, as a score change of 8 to 10 points is considered to be clinically relevant (on a 0–100 scale) [56]. A total sample size of 118 patients was planned (59 patients per group), as we expected a discontinuation rate of 20% (47 patients per group needed when there is no discontinuation). As the sample size calculation was performed before the start of the study (only limited information available) we could not account for correlation within subjects. The intention-to-treat principle (ITT) was used for all primary and secondary analyses, so patients were analyzed in the group they were allocated to despite protocol violations like discontinuation of treatment. Sub-analyses were conducted for hip and knee patients separately. For the primary and secondary endpoints mixed-model repeated measures (MMRM) were used. The model included the fixed categorical effect of treatment, visit and joint (=stratification factor). Two interaction factors were included, namely the treatment-by-visit interaction and the joint-by-treatment interaction. An MMRM was also used to detect any effect on PPT (joint and remote location) during duloxetine treatment (so only patients randomized to the duloxetine intervention group were used for this analysis). This model included the fixed categorical effect of visit, with a pairwise comparison of the different visits (time points). A *p*-value < 0.05 was considered statistically significant.

## Results

In total 3402 patients were screened; 725 were eligible and were asked to participate, and 112 of them indicated wanting to participate in the study (Fig. 1). Patients who declined to participate did not differ on mean mPDQ score (*p* = 0.999) and were equal with respect to hip/knee ratio (*p* = 0.184). However, they were on average older than participating patients (difference: 5.2 year; *p* < 0.0001). More females declined to participate (72% females in the non-randomized group vs. 62% in the randomized group; *p* = 0.031). There was one screen failure due to low sodium so the definitive study population consisted of 111 patients, 57 of them randomized to the duloxetine intervention group and 54 to the care-as-usual group (see the CONSORT Flow Diagram, Fig. 1). Baseline characteristics were comparable between the duloxetine intervention and the care-as-usual group (see Table 1). Knee OA patients comprised 55% of the study group. Mean age of the whole study group was 62.7 years and the majority were female (62.2%) and overweight (mean BMI: 28.9). There were more smokers in the duloxetine intervention group (*p* = 0.053). Joint-specific sub-analyses revealed that 58% of the duloxetine intervention patients in the hip group were smokers, versus 25.8% in the knee OA group. In Table 1 characteristics and baseline variables are listed.

**Table 1:** Characteristics and outcome variables of study participants at baseline (T0)^a^

Characteristics	Duloxetine intervention ***N*** = 57	Care-as-usual ***N*** = 54	
**Joint**			
Knee	54.4 (31)	55.6 (30)	
Hip	52.0 (26)	48.0 (24)	
**Age** (years), mean ± SD	61.5 ± 8.1	64 ± 8.7	
**Female**	66.7 (38)	57.4 (31)	
**BMI** (kg/m^2^), mean ± SD	28.8 ± 4.9	29 ± 3.9	
**Cohabitation** (yes)	77.2 (44)	75.9 (41)	
**Educational level**			
Higher	40.4 (23)	38.9 (21)	
Secondary	50.9 (29)	55.6 (30)	
No or lower	8.8 (5)	5.6 (3)	
**Smoking** (yes)	26.3 (15)	11.1 (6)	
**ASA Classification**			
I	33.3 (19)	27.8 (15)	
II	54.4 (31)	68.5 (37)	
III	12.3 (7)	3.7 (2)	
**Comorbidities (/9)**, median (Q1;Q3)	1 (0;1)	1 (0;2)	
Back disorder	7 (4)	16.7 (9)	
Diabetes	5.3 (3)	11.1 (6)	
Cancer	–	1.9 (1)	
Chronic bowel disorder	8.8 (5)	9.3 (5)	
Migraine	8.8 (5)	9.3 (5)	
Cardiopulmonary condition (/4)	0 (0;1)	0 (0;1)	
**PCS (0–52),** mean ± SD	15.5 (9.5)	17.4 (10.6)	
**HADS-A (0–21)**, median (Q1;Q3)	3 (1;5)	3 (1;5)	
**HADS-D (0–21)**, median (Q1;Q3)	3 (2;5)	3 (2;5)	
**Number of painful body regions/joints** (/20), median (Q1;Q3)	2 (1;3.5)	2 (1;4)	
**Duration of osteoarthritis pain** (months), median (Q1;Q3)	48 (22.5;90.0)	36 (16;7.75.0)	
**KL grade**			
KL grade II	14 (8)	27.8 (15)	
KL grade III	78.9 (45)	68.5 (37)	
KL grade IV	7 (4)	3.7 (2)	
**History of surgery in index joint**	52.6 (30)	53.7 (29)	
**Analgesic injection in index joint (past year)**	24.6 (14)	27.8 (15)	
**Analgesic usage in past week**	64.9 (37)	70.4 (38)	
Acetaminophen	45.6 (26)	48.1 (26)	
Nonsteroidal anti-inflammatory drugs	36.8 (21)	27.8 (15)	
Weak opioids	3.5 (2)	5.6 (3)	
Strong opioids	1.8 (1)	–	
Others	–	–	
**KOOS/HOOS (0–100)**			
ain (mean ± SD)	38.6 ± 14.1	30.9 ± 12.7	
Symptoms (mean ± SD)	43.4 ± 18.7	41.1 ± 14.6	
ADL (mean ± SD)	41.7 ± 15.2	38.6 ± 14.6	
QOL (mean ± SD)	25.4 ± 13.8	21.4 ± 12.8	
**mPDQ**	15.5 ± 4.7	16 ± 4.6	
PPT-Joint, median (Q1;Q3)	3.9 (2.3;4.7)	4.3 (1.9;2.2)	
PPT-Remote, median (Q1;Q3)	3.2 (1.8;4.3)	3.0 (2.2;3.8)	
**VAS-past week**			
VAS-Rest (mean ± SD)	46.6 ± 24.8	58.7 ± 18.2	
VAS-Movement (mean ± SD)	68.1 ± 15.7	71.1 ± 17.2	

^a^Except where indicated otherwise, values are presented as % (n)

*BMI* body mass index, *ASA* American Society of Anesthesiologists, *PCS* Pain Catastrophizing Scale, *HADS-A* Hospital Anxiety and Depression Scale, anxiety subscale, *HADS-D* Hospital Anxiety and Depression Scale, depression subscale, *KL* Kellgren & Lawrence, *KOOS/HOOS* Knee injury and Osteoarthritis Outcome Score/Hip disability and Osteoarthritis Outcome Score, *mPDQ* modified painDETECT questionnaire, *PPT* pressure pain threshold, *VAS* Visual Analogue Scale

### Functional status and quality of life (KOOS/HOOS)

**Fig. 2:**
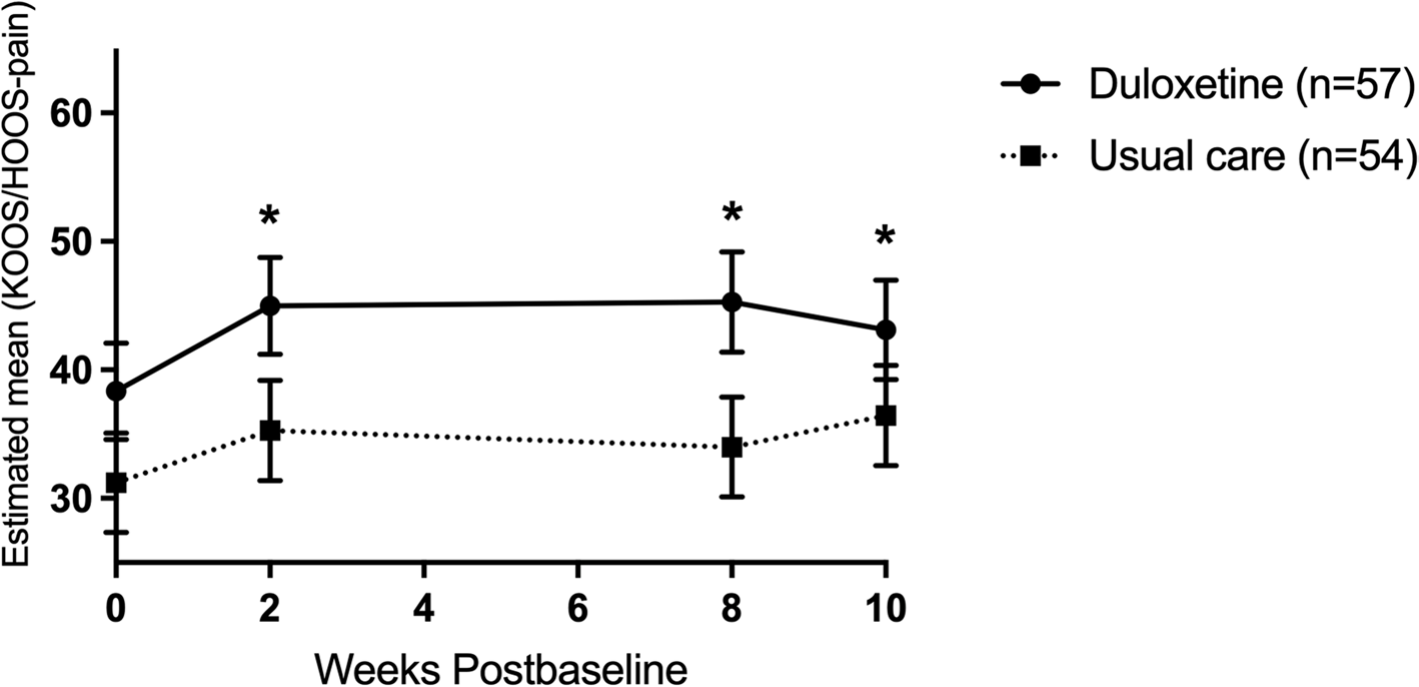
Total group: Change in the adjusted mean score of the pain subscale of the KOOS/HOOS (based on estimates from model). Abbreviations: *n* = number of randomized patients with non-missing data at baseline

At the end of the treatment phase (time point T2), duloxetine intervention patients had statistically significantly higher scores (better) than the care-as-usual group on the domains of pain, symptoms and ADL (see Fig. 2 and Table 2). Adjusted mean differences between the duloxetine intervention and the care-as-usual group ranged from 9.2 to 11.3 points (see Table 2). No differences were observed in the QOL subscale. The subscale sport and recreation could not be used due to a high number of missing items. Joint-specific sub-analyses revealed that only knee OA patients in the duloxetine intervention group scored statistically significantly higher within these domains compared to the care-as-usual group. Adjusted mean differences in knee OA patients for these subscales at T2 ranged from 17.0 to 19.3 points (see Tables 1 and 2 in the appendices).

**Table 2:** Estimated means from models at T2 and T3

	Scale range	Duloxetine intervention	Care-as-usual	Adjusted mean difference	***P***-value
T2 (after treatment phase)					
**KOOS/HOOS**					
Pain	0–100	45.3 (41.4, 49.2)	34.0 (30.1, 37.9)	11.3 (5.8, 16.8)	**< 0.001**
Symptoms	0–100	49.1 (44.7, 53.5)	39.9 (35.5, 44.3)	9.2 (3.0, 15.4)	**0.004**
ADL	0–100	47.8 (43.5, 52.1)	37.3 (33.0, 41.6)	10.5 (4.5, 16.6)	**0.001**
QOL	0–100	26.8 (22.8, 30.8)	22.3 (18.3, 26.4)	4.5 (0.5, 12.3)	0.124
**mPDQ**	−1-38	11.7 (10.3, 13.2)	15.4 (13.9, 16.8)	3.6 (1.6, 5.7)	**0.001**
**VAS-past week**					
VAS-Rest	0–100	40.8 (34.8, 46.8)	57.8 (51.8, 63.8)	17.0 (8.5, 25.5)	**< 0.001**
VAS-Movement	0–100	53.9 (48.9, 59.1)	70.6 (65.6, 75.7)	16.7 (9.5, 23.9)	**< 0.001**
**PGI-I**^a^	1–7	3.3 ± 1.7	5.0 ± 0.9	1.77 (1.2, 2.3)	**< 0.001**
Much or very much better#		43.8% (21/48)	0% (0/51)	–	**< 0.001**
Much or very much worse#		12.5% (6/48)	33.3% (17/51)	–	**0.018**
T3 (after tapering phase)					
**KOOS/HOOS**					
Pain	0–100	43.1 (39.2, 47.0)	36.4 (32.6, 40.3)	6.7 (1.2, 12.1)	**0.017**
Symptoms	0–100	44.8 (40.5, 49.2)	41.7 (37.4, 46.1)	3.1 (−2.8, 10.5)	0.325
ADL	0–100	45.5 (41.2, 49.7)	40.2 (36.0, 44.5)	5.2 (−0.8, 11.2)	0.089
QOL	0–100	27.0 (23.0, 31.0)	22.3 (18.3, 26.4)	4.7 (−1.0, 10.4)	0.105
**mPDQ**	−1-38	13.0 (11.6, 14.4)	15.1 (13.7, 16.6)	2.1 (0.1, 4.2)	**0.04**
**VAS-past week**					
VAS-Rest	0–100	42.6 (36.6, 48.6)	60.3 (54.3, 66.3)	17.7 (9.3, 26.2)	**< 0.001**
VAS-Movement	0–100	58.8 (53.8, 63.8)	69.2 (64.2, 74.3)	10.4 (3.3, 17.6)	**0.004**
**PGI-I**^**a**^	1–7	4.0 ± 1.6	5.2 ± 1.1	1.2 (0.7, 1.7)	**< 0.001**
Much or very much better#	.	22.5% (11/49)	0% (0/51)	–	**< 0.001**
Much or very much worse#		22.5% (11/49)	51.0% (26/51)	–	**0.004**

^a^observed values; # % (n/N)

**Fig. 3:**
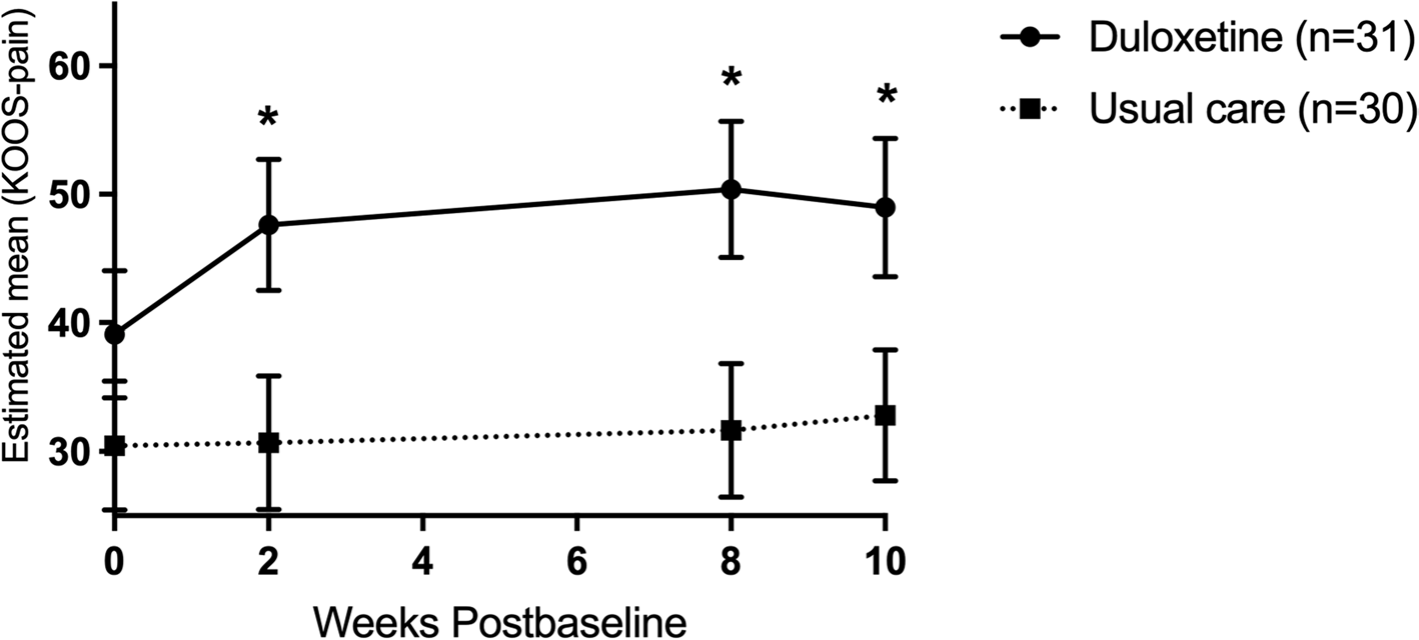
Knee OA group: Change in the adjusted mean score of the pain subscale of the KOOS (based on estimates from model). Abbreviations: *n* = number of randomized patients with non-missing data at -baseline

**Fig. 4:**
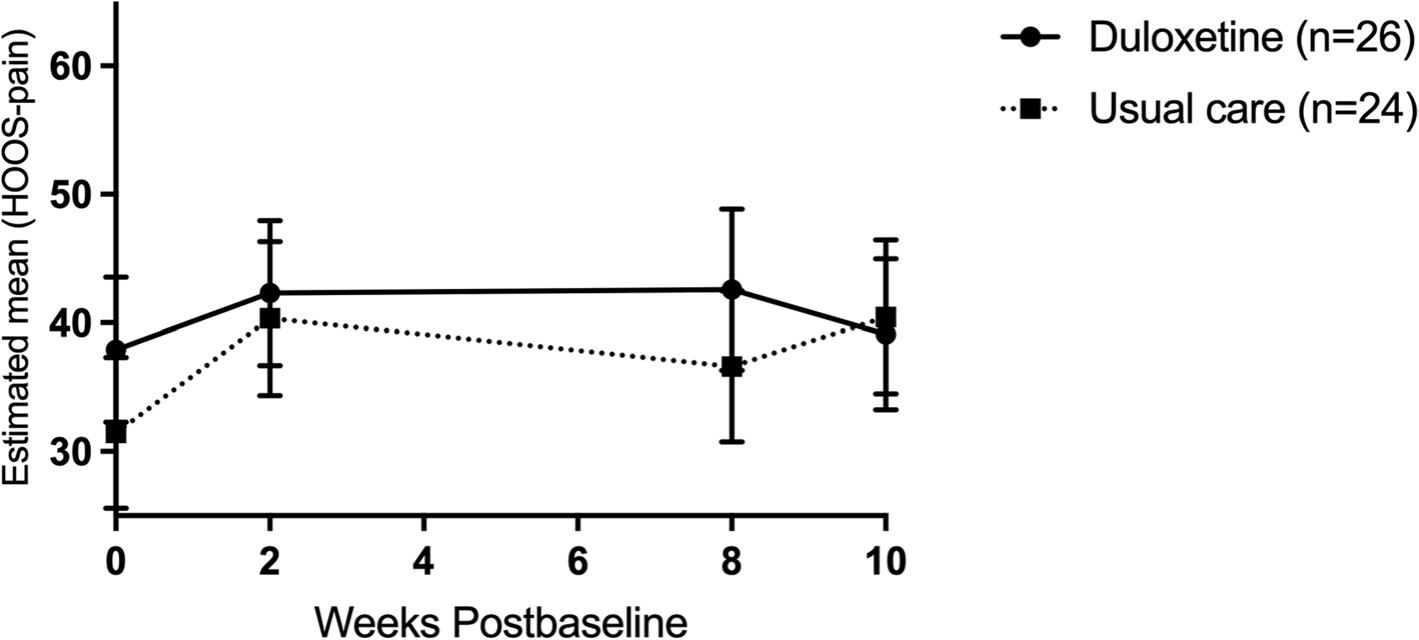
Hip OA group: Change in the adjusted mean score of the pain subscale of the HOOS (based on estimates from model). Abbreviations: *n* = number of randomized patients with non-missing data at baseline

After the tapering phase (time point T3) only the statistically significant difference in the KOOS/HOOS pain domain was preserved (6.7 points), no statistically significant differences were apparent anymore in the KOOS/HOOS domains of symptoms and ADL (see Table 2). Interestingly, joint-specific sub-analyses revealed that next to the pain subscale (Fig. 3), duloxetine intervention patients in the knee OA group scored statistically significantly higher on the symptoms, ADL and QOL subscales at time point T3. Adjusted mean differences on these subscales ranged from 10.2 to 16.2 points (see Table 3 in the appendices). No statistically significant differences were seen in hip OA patients (Fig. 4 and Table 4 in the appendices).

### Neuropathic-like symptoms

mPDQ scores in the duloxetine intervention group were statistically significantly lower at time points T2 and T3 compared to the care-as-usual group. The difference between the duloxetine and the care-as-usual group was the biggest at time point T2 (3.6 vs. 2.1 points at T3, see Table 2). In joint-specific analyses, knee OA patients who used duloxetine had statistically significantly lower mPDQ scores at time points T2 (5.1 points lower, *p* = 0.002) and T3 (4.1 points lower, *p* = 0.012). By contrast, among hip OA patients no difference was observed between the duloxetine intervention and the care-as-usual group at any time point (see Tables 1, 2, 3 and 4 in the appendices).

### Pressure pain sensitization

A pairwise comparison of the different time points in the duloxetine intervention group did not find any statistically significant differences at the two test sides (joint and wrist area). Joint-specific analyses did not find any changes between PPTs during treatment either (see Table 3).

**Table 3:** Pressure pain sensitization outcomes at T2 and T3^a^

	T0	T2	***p***-value (T0-T2)	T3	***p***-value (T0-T3)
**Total group**					
Joint	3.9 (3.4, 4.5)	4.2 (3.6, 4.8)	0.620	3.9 (3.3, 4.6)	0.849
Remote	3.4 (2.8, 3.9)	3.6 (3.0, 4.3)	0.554	3.86 (3.2, 4.5)	0.249
**Knee**					
Joint	3.9 (3.0, 4.7)	3.9 (3.1, 4.8)	0.896	3.8 (2.9, 4.7)	0.964
Remote	3.3 (2.4, 4.1)	3.7 (2.8, 4.7)	0.478	4.1 (3.1, 5.1)	0.220
**Hip**					
Joint	4.1 (3.2, 4.9)	4.5 (3.5, 5.5)	0.480	4.0 (3.0, 5.0)	0.972
Remote	3.5 (2.8, 4.2)	3.5 (2.6, 4.3)	0.999	3.6 (2.8, 4.4)	0.806

^a^Adjusted mean value of peak force in kg (95% CI); Missing data range: 8.7–9.6%; Post hoc Bonferroni correction was performed, results were not significantly different

### Pain during the past week

Pain intensity at T2 and T3 was lower in the duloxetine intervention group than in the care-as-usual group (Table 2). The biggest difference between the two groups was observed for pain at rest (difference VAS-Rest: 17.0 [T2]; 17.7 [T3]). The effect on pain in movement shrunk after duloxetine tapering (T3) (difference VAS-Movement: 16.7 [T2]; 10.4 [T3]). Joint-specific sub-analyses revealed statistically significant differences only between the duloxetine intervention and the care-as-usual group among the knee OA patients (see Tables 1, 2, 3 and 4 in the appendices).

### Patient global impression of improvement (PGI-I)

Subjective overall improvement was most prominent at time point T2, with 44.75% of patients in the duloxetine intervention group feeling that their joint complaints were much or very much better. At T3 this percentage was lower, at 22.45%. However, none of the patients in the care-as-usual group experienced any improvement at any time point (Table 2). Joint-specific sub-analyses showed a comparable distribution (see Tables 1, 2, 3, and 4 in the appendices).

### Safety measures

Nearly 95% of patients who enrolled in the duloxetine intervention experienced an AE; 21% quit due to an AE. A median of three AEs were experienced by duloxetine users. Most common AEs reported were headache (33%), somnolence (30%), nausea (28%) and dry mouth (28%). Other reported AEs are shown in Table 4.

**Table 4:** Adverse events experienced by the duloxetine treatment group

	Duloxetine intervention ***N*** = 57 – n (%)*
Experienced an AE	54 (94.7%)
Discontinued due to AE(s)	12 (21.1%)
Number of AEs per patient, median (Q1;Q3)	3 (2;5)
AEs in ≥5% of patients	
Headache	19 (33.3%)
Somnolence	17 (29.8%)
Nausea	16 (28.1%)
Dry mouth	16 (28.1%)
Constipation	12 (21.1%)
Fatigue	10 (17.5%)
Dizziness	10 (17.5%)
Insomnia	7 (12.3%)
Hyperhidrosis	6 (10.5%)
Paresthesia	6 (10.5%)
Diarrhea	4 (7.0%)
Dyspepsia	4 (7.0%)
Dysuria	4 (7.0%)
Hot flushes	4 (7.0%)
Vomiting	4 (7.0%)
Palpations	3 (5.3%)
Blurred vision	3 (5.3%)
Musculoskeletal pain	3 (5.3%)

*Unless stated otherwise. Other reported AEs: 3.5% (*n* = 2) of patients experienced abnormal dreams, dysgeusia, abdominal pain flatulence, abnormal orgasm, erectile dysfunction, abnormal urine odor, polyuria, muscle spasm and nocturia. 1.8% (*n* = 1) of patients experienced apathy, rigors, night sweats, tinnitus, tension, orthostatic hypotension, decreased libido, mood swings, cough, decreased appetite and elevated blood pressure

### Missing data

The amount of missing data in the models used (MMRM) ranged from 3.6 to 10.8% (range T1-T3). For details see Table 5.

**Table 5:** Missing values for total group (*n* = 111)

	KOOS / HOOS Pain	Symptoms	ADL	QOL	mPDQ	VAS-Rest	VAS-Movement
**Baseline n (%)**	0 (0)	0 (0)	0 (0)	0 (0)	0 (0)	1 (0.9)	0 (0)
**T 1 n (% missing)**	5 (4.5)	4 (3.6)	4 (3.6)	4 (3.6)	6 (5.4)	4 (3.6)	4 (3.6)
**T 2 n (% missing)**	11 (9.9)	11 (9.9)	11 (9.9)	11 (9.9)	12 (10.8)	12 (10.8)	12 (10.8)
**T 3 n (% missing)**	9 (8.1)	9 (8.1)	9 (8.1)	10 (9.0)	10 (9.0)	9 (8.1)	9 (8.1)

*n* number of cases missing, % percentage missing

## Discussion

In this pragmatic enriched randomized controlled trial among end-stage hip and knee OA patients, an 8-week duloxetine intervention showed having more analgesic effects than usual care. Nearly 44% of patients felt much to very much better after 8 weeks of duloxetine usage compared to 0% in the usual care group. The duloxetine intervention seems to have a clinically relevant effect in improving joint related pain and function (KOOS/HOOS Pain, Symptoms and ADL domain) [56, 57]. Additional separate sub-analyses revealed that these observed effects in the total study group were likely explained by relief of pain and symptoms in knee OA patients who used duloxetine. Clinically relevant and statistically significant effects of the duloxetine intervention were namely only observed among knee OA patients. However, just like knee OA patients, hip OA patients did experience subjective improvement (on the PGI-I) of their symptoms. Nearly 43% of patients felt much to very much better after 8 weeks of duloxetine usage, compared to 0% in the usual care group.

The two-week tapering phase which included 2 weeks on half the treatment phase dosage showed decreased analgesic effects of duloxetine. None of the differences within the pain- and function-related outcome measures on the KOOS/HOOS reached clinically relevant threshold levels [56, 57]. Joint-specific sub-analyses did reveal that in the knee OA patients mean differences between the two groups were still statistically significant and clinically relevant for nearly all outcome measures, including pain-related ones [56, 57]. The perceived level of improvement (much to very much) after tapering (on the PGI-I) did shrink from 43% to around 20% in both hip and knee OA patients.

The present results can be compared to previous literature only to a limited extent. Firstly, studies among hip OA patients are lacking. Secondly, other knee OA studies as well as studies on other chronic musculoskeletal pain conditions compared duloxetine intervention to placebo in a highly controlled fashion, as opposed to our pragmatic design (care-as-usual control group). Lastly, none of the studies used an enriched design that only included patients with end-stage OA pain with neuropathic features (as a sign of a centralized pain component [CS]). Despite these issues of heterogeneity, results found in previous studies were comparable to ours. Those studies found the duloxetine intervention group to be superior in relieving OA-related pain when compared to the care-as-usual group [20, 52, 58]. The present study also found a similar proportion of patients who indicated that their joint complaints were much to very much better after taking duloxetine [20]. These results were somewhat unexpected, as we hypothesized that our enriched population should react better to duloxetine.

The discrepancy between knee and hip OA regarding the effect of the duloxetine intervention is clearly noticeable, yet one must bear in mind that the study was not powered on detecting statistically significant differences within the two OA entities separately. On the other hand, nearly all differences in the knee OA group were statistically significant and clinically relevant. One could only ponder about the reasons why these differences are so prominent between the two OA entities. One factor could be that knee OA patients are more centrally sensitized and thus more prone to respond to a centrally acting agent like duloxetine. This could be the result of enhanced pain perception and proprioception in knee OA, which provokes early symptomatic presentation [59]. As a consequence, knee OA patients experience more long-lasting pain, which could sensitize the peripheral and later the central nervous system (CS) [6]. Another explanation for the found discrepancies could lie in the reduced bioavailability of duloxetine due to smoking [60]. Smoking causes an increase in the expression of CYP1A2, which is associated with a one-third decrease in the bioavailability of duloxetine [60]. This could have had its effects in the relatively small hip OA sample, as the majority of duloxetine users where smokers. Larger studies, adequately powered for each joint, are needed to draw conclusions: a larger study could provide more insight into the effect of a duloxetine intervention in a subgroup of patients who experience more neuropathic-like symptoms (higher mPDQ scores).

Nearly all patients who used duloxetine experienced treatment-emergent AEs (94.7%). These were generally modest and mainly present at the start of the duloxetine intervention. The found percentage is higher than reported percentages in literature though: a recent study by Wang et al. found that 60.8% of duloxetine patients experienced at least one treatment-emergent AE when using 60 mg/d duloxetine for 13 weeks [20]. Compared to literature, the number of reported duloxetine AEs is higher in the present study [60]: a significant portion of the study patients complained of headache (33.3%), whereas the literature reports this for only 13% of patients. Equally for somnolence, our study reported nearly 30% whereas previous studies reported only 12%. These discrepancies could be the result of the way AEs were recorded. Patients in the present study reported their feelings and side effects in a diary, which forced them to think thoroughly about side effects on a daily basis. The discontinuation rate due to treatment-emergent AEs was consistent with percentages presented in literature, 16.3% versus 21.1% in the present study [60].

Due to its uniqueness, by using a pragmatic and enriched trial design this study offers new insights. The foremost insight is that end-stage knee OA patients still possess the ability to respond well to conservative duloxetine treatment. Significant pain relief was already noticeable after 2 weeks of treatment. As a result it could be hypothesized that joint-replacement surgery can be postponed in a subset of patients, or could even no longer be needed. This would have a major impact not only at the individual level but also at the societal level, as joint replacement surgery is a costly and frequently performed intervention worldwide. Another new insight is that it appears possible to desensitize knee OA patients preoperatively. Clinically relevant pain relief up to 10 weeks was seen in knee OA patients. It is known that a preoperative level of central sensitization (CS) is linked with less pain relief after arthroplasty, thus even when articular nerve fibers that induced CS are removed [6]. This could imply that preoperative pain desensitization would lower the risk of experiencing residual postsurgical pain. Our present population will therefore be followed up to evaluate the postoperative effects of preoperative desensitization. This is highly interesting, as residual postsurgical pain is present in about 29% of TKA patients [61].

Despite the advantages, the design used also has several limitations. Due to its enriched nature effects could only be seen in end-stage OA patients. However, results will probably not be worse in non-end-stage OA patients with neuropathic-like symptoms, as we believe that these patients should be easier to desensitize because of their less prolonged exposure to pain. Another limitation could be the relatively high proportion of patients who declined to participate (85%). The study patients and the patients who declined differed in age and gender distribution, so a level of selection bias could be present. It seems to be of little importance though, as the patients who declined did not differ on mean mPDQ-score from those patients who participated. As duloxetine therapy is contraindicated for subjects with certain comorbidities, our results cannot be extrapolated to every patient with end-stage OA and neuropathic-like symptoms. Nearly 20% of our screened target population was deemed as not suitable due to duloxetine-related contraindications. On the other hand, other analgesics often used in OA, such as NSAIDs, are also frequently contraindicated.

## Conclusions

Adding duloxetine treatment to usual care seems to be especially beneficial for end-stage knee OA patients with neuropathic-like symptoms/central sensitization. End-stage hip OA patients seem to be non-responsive to duloxetine, so there seems to be room for additional conservative treatment in a subset of these patients. It could therefore be advised to screen every knee arthroplasty candidate for the possibility of adding duloxetine to usual care. Treatment should be done in a controlled fashion, as AEs are commonly experienced. In knee OA patients treatment seems to have the potential to delay the process toward arthroplasty. In the long term this could even mean fewer joint replacement surgeries and hence revisions. Future postoperative results of our study should be awaited to have further information on the ability to reduce residual postsurgical pain.

## Supplementary Information

**Additional file 1.**

## Data Availability

The datasets used and/or analysed during the current study are available from the corresponding author on reasonable request.

## Abbreviations

OA: Osteoarthritis
RCT: Randomized Controlled Trial
CS: Central sensitization
KOOS: Knee injury and Osteoarthritis Outcome Score
HOOS: Hip disability and Osteoarthritis Outcome Score
NSAIDs: Nonsteroidal anti-inflammatory drugs
DOA: Duloxetine in OsteoArthritis
UMCG: University Medical Center Groningen
THA: Total hip arthroplasty
TKA: Total knee arthroplasty
mPDQ: Modified painDETECT
METc: Medical Ethics Committee
GCP: Good Clinical Practice
NTR: Netherlands Trial Registry
BMI: Body mass index
ASA: American Society of Anesthesiologists
PCS: Pain Catastrophizing Scale
HADS: Hospital Anxiety and Depression Scale
KL: Kellgren and Lawrence
AE: Adverse event
PPT: Pressure pain thresholds
VAS: Visual Analogue Scale
PGI-I: Patient Global Impression of Improvement
MMRM: Mixed-model repeated measures
