# Supplementary material for: Duloxetine in OsteoArthritis (DOA) study: effects of duloxetine on pain and function in end-stage hip and knee OA – a pragmatic enriched randomized controlled trial
Source: BMC Musculoskelet Disord. 2022 Feb 5;23:115. doi: 10.1186/s12891-022-05034-0 (PMC8818142; doi:10.1186/s12891-022-05034-0)
Supplement: Supplementary file 1 — Additional file 1. [file 12891_2022_5034_MOESM1_ESM.docx]

**Appendices**

**Supplementary table 1.** Estimated means from models at T2 for the Knee OA group

|  | **Scale range** | **Duloxetine**  **intervention** | **Care-as-usual** | **Adjusted mean difference** | **P-value** |
| --- | --- | --- | --- | --- | --- |
| **KOOS** |  |  |  |  |  |
| Pain | 0-100 | 50.4 (45.1, 55.7) | 31.7 (26.5, 36.8) | 18.7 (11.3, 26.1) | **<0.001** |
| Symptoms | 0-100 | 60.5 (53.9, 67.0) | 43.5 (37.1, 49.9) | 17.0 (7.8, 26.1) | **<0.001** |
| ADL | 0-100 | 55.3 (49.5, 61.2) | 36.1 (30.3, 41.8) | 19.3 (11.0, 27.5) | **<0.001** |
| QOL | 0-100 | 30.9 (25.4, 36.3) | 22.5 (17.2, 27.9) | 8.3 (0.7, 15.9) | **0.033** |
| **mPDQ** | -1-38 | 11.1 (8.8, 13.4) | 16.2 (14.0, 18.4) | 5.1 (1.9, 8.3) | **0.002** |
| **VAS-past week** |  |  |  |  |  |
| VAS-Rest | 0-100 | 35.4 (27.2, 43.5) | 59.7 (51.5, 67.9) | 24.3 (12.8, 35.9) | **<0.001** |
| VAS-Movement | 0-100 | 51.0 (43.9, 58.2) | 75.3 (68.2, 82.5) | 24.3 (14.2, 34.4) | **<0.001** |
| **PGI-I*** | 1-7 | 3.2 ± 1.6 | 5.0 ± 1.0 | 1.8 (1.1, 2.6) | **<0.001** |
| Much or very much better^#^ |  | 44.4% (12/27) | 0.0% (0/28) | - | **<0.001** |
| Much or very much worse^#^ |  | 11.1% (3/27) | 35.7% (10/28) | - | 0.055 |
| * observed values; # % (n/N) | | | | | |

**Supplementary table 2.** Estimated means from models at T2 for the Hip OA group

|  | **Scale range** | **Duloxetine**  **intervention** | **Care-as-usual** | **Adjusted mean difference** | **P-value** |
| --- | --- | --- | --- | --- | --- |
| **HOOS** |  |  |  |  |  |
| Pain | 0-100 | 42.6 (36.3, 48.9) | 36.6 (30.8, 42.5) | 6.0 (-2.6, 14.5) | 0.172 |
| Symptoms | 0-100 | 40.2 (34.1, 46.3) | 36.3 (30.5, 41.9) | 4.0 (-4.4, 12.3) | 0.347 |
| ADL | 0-100 | 43.4 (36.6, 50.2) | 38.1 (31.7, 44.5) | 5.3 (-4.0, 14.6) | 0.264 |
| QOL | 0-100 | 25.6 (18.9, 32.3) | 21.6 (15.4, 27.9) | 4.0 (-5.2, 13.1) | 0.393 |
| **mPDQ** | -1-38 | 12.2 (10.4, 14.1) | 14.4 (12.6, 16.1) | 2.1 (-0.4, 4.7) | 0.098 |
| **VAS-past week** |  |  |  |  |  |
| VAS-Rest | 0-100 | 45.4 (35.7, 55.1) | 55.0 (46.0, 64.0) | 9.6 (-3.6, 22.9) | 0.153 |
| VAS-Movement | 0-100 | 56.8 (49.0, 64.6) | 65.5 (58.2, 72.8) | 8.7 (-2.0, 19.4) | 0.112 |
| **PGI-I*** | 1-7 | 3.3 ± 1.7 | 5.0 ± 0.8 | 1.7 (0.9, 2.5) | **<0.001** |
| Much or very much better^#^ |  | 42.9% (9/21) | 0.0% (0/23) | - | **<0.001** |
| Much or very much worse^#^ |  | 14.3% (3/21) | 30.4% (7/23) | - | 0.287 |
| * observed values; # % (n/N) | | | | | |

**Supplementary table 3.** Estimated means from models at T3 for the Knee OA group

|  | **Scale range** | **Duloxetine**  **intervention** | **Care-as-usual** | **Adjusted mean difference** | **P-value** |
| --- | --- | --- | --- | --- | --- |
| **KOOS** |  |  |  |  |  |
| Pain | 0-100 | 49.0 (43.6, 54.4) | 32.8 (27.7, 37.9) | 16.2 (8.7, 23.6) | **<0.001** |
| Symptoms | 0-100 | 54.7 (48.0, 61.4) | 44.5 (38.1, 50.8) | 10.2 (1.0, 19.4) | **0.030** |
| ADL | 0-100 | 54.4 (48.4, 60.4) | 40.3 (34.6, 46.0) | 14.1 (5.9, 22.4) | **0.001** |
| QOL | 0-100 | 32.2 (26.7, 37.8) | 21.4 (16.1, 26.8) | 10.8 (3.1, 18.5) | **0.006** |
| **mPDQ** | -1-38 | 12.0 (9.7, 14.3) | 16.1 (13.9, 18.4) | 4.1 (0.9, 7.3) | **0.012** |
| **VAS-past week** |  |  |  |  |  |
| VAS-Rest | 0-100 | 36.5 (28.2, 44.8) | 64.3 (56.5, 72.2) | 27.8 (16.4, 39.3) | **<0.001** |
| VAS-Movement | 0-100 | 54.2 (46.9, 61.5) | 74.3 (67.4, 81.2) | 20.1 (10.1, 30.1) | **<0.001** |
| **PGI-I*** | 1-7 | 3.9 ± 1.5 | 5.2 ± 1.2 | 1.3 (0.6, 2.1) | **<0.001** |
| Much or very much better^#^ |  | 23.1% (6/26) | 0.0% (0/28) | - | **0.009** |
| Much or very much worse^#^ |  | 19.2% (5/26) | 50% (14/28) | - | **0.024** |
| * observed values; # % (n/N) | | | | | |

**Supplementary table 4.** Estimated means from models at T3 for the Hip OA group

|  | **Scale range** | **Duloxetine intervention** | **Care-as-usual** | **Adjusted mean difference** | **P-value** |
| --- | --- | --- | --- | --- | --- |
| **HOOS** |  |  |  |  |  |
| Pain | 0-100 | 39.1 (33.3, 45.0) | 40.5 (34.5, 46.4) | -1.3 (-9.7, 7.0) | 0.753 |
| Symptoms | 0-100 | 36.5 (30.8, 42.2) | 39.1 (33.3, 45.0) | -2.7 (-10.8, 5.5) | 0.519 |
| ADL | 0-100 | 38.9 (32.6, 45.3) | 40.3 (33.8, 46.8) | -1.4 (-10.5, 7.8) | 0.769 |
| QOL | 0-100 | 23.7 (17.4, 30.0) | 22.3 (15.9, 28.7) | 1.4 (-7.5, 10.4) | 0.755 |
| **mPDQ** | -1-38 | 14.0 (12.3, 15.8) | 14.1 (12.3, 15.9) | -0.1 (-2.6, 2.3) | 0.918 |
| **VAS-past week** |  |  |  |  |  |
| VAS-Rest | 0-100 | 47.2 (38.2, 56.3) | 55.7 (46.5, 65.0) | 8.5 (-4.4, 21.5) | 0.195 |
| VAS-Movement | 0-100 | 63.5 (56.2, 70.9) | 63.6 (56.1, 71.1) | 0.1 (-10.4, 10.5) | 0.990 |
| **PGI-I*** | 1-7 | 4.3 ± 1.7 | 5.3 ± 1.1 | 1.0 (0.2, 1.9) | **0.015** |
| Much or very much better^#^ |  | 21.7% (5/23) | 0.0% (0/23) | - | **0.049** |
| Much or very much worse^#^ |  | 26.1% (6/23) | 52.2% (12/23) | - | 0.130 |
| * observed values; # % (n/N) | | | | | |
